# Myeloid Deletion of SIRT1 Aggravates Serum Transfer Arthritis in Mice via Nuclear Factor-κB Activation

**DOI:** 10.1371/journal.pone.0087733

**Published:** 2014-02-03

**Authors:** Young-Sool Hah, Yun-Hong Cheon, Hye Song Lim, Hee Young Cho, Byung-Hyun Park, Sun-O Ka, Young-Rae Lee, Dong-Won Jeong, Hyun-Ok Kim, Myung-Kwan Han, Sang-Il Lee

**Affiliations:** 1 Clinical Research Institute, Gyeongsang National University Hospital, Jinju, Republic of Korea; 2 Department of Internal Medicine and Institute of Health Science, Gyeongsang National University School of Medicine, Jinju, Republic of Korea; 3 Department of Biochemistry, Chonbuk National University Medical School, Jeonju, Republic of Korea; 4 Department of Oral biochmistry, Wonkwang University School of Dentistry, Iksan, Republic of Korea; 5 Department of Microbiology, Chonbuk National University Medical School, Republic of Korea; Northwestern University Feinberg School of Medicine, United States of America

## Abstract

**Objective:**

SIRT1 modulates the acetylation of the p65 subunit of nuclear factor-κB (NF-κB) and plays a pivotal role in the inflammatory response. This study sought to assess the role of SIRT1 in rheumatoid arthritis (RA) using a myeloid cell-specific SIRT1 knockout (mSIRT1 KO) mouse.

**Methods:**

mSIRT1 KO mice were generated using the loxP/Cre recombinase system. K/BxN serum transfer arthritis was induced in mSIRT1 KO mice and age-matched littermate loxP control mice. Arthritis severity was assessed by clinical and pathological scoring. The levels of inflammatory cytokines in the serum and joints were measured by ELISA. Migration, M1 polarization, cytokine production, osteoclastogenesis, and p65 acetylation were assessed in bone marrow-derived monocytes/macrophages (BMMs).

**Results:**

mSIRT1 KO mice showed more severe inflammatory arthritis and aggravated pathological findings than control mice. These effects were paralleled by increases in IL-1, TNF-α, TRAP-positive osteoclasts, and F4/80^+^ macrophages in the ankles of mSIRT1 KO mice. In addition, BMMs from mSIRT1 KO mice displayed hyperacetylated p65 and increased NF-κB binding activity when compared to control mice, which resulted in increased M1 polarization, migration, pro-inflammatory cytokine production, and osteoclastogenesis.

**Conclusion:**

Our study provides *in vivo* evidence that myeloid cell-specific deletion of SIRT1 exacerbates inflammatory arthritis via the hyperactivation of NF-κB signaling, which suggests that SIRT1 activation may be beneficial in the treatment of inflammatory arthritis.

## Introduction

The mammalian sirtuin members, named SIRT1 to SIRT7, are class III histone deacetylases that regulate senescence, stress resistance, metabolism, and inflammation [1], [2]. In particular, SIRT1 has been demonstrated to be a pivotal molecule in the modulation of inflammation through the deacetylation of histones and non-histone proteins [3]. However, there are limited and controversial data regarding the roles of SIRTs in rheumatoid arthritis (RA). Niederer *et al*. showed that TNF-α-induced over-expression of SIRT1 contributes to chronic inflammation by increasing proinflammatory cytokines and inhibiting apoptosis in RA synovial cells [4]. Subsequently, the same authors reported that SIRT6 attenuates cigarette smoke- and TNF-α-induced matrix metalloproteinase-1 (MMP1) production in RA fibroblast-like synoviocytes (RA-FLS) [5]. More recently, we showed that SIRT6 over-expression effectively reduces the inflammatory response in RA-FLS and the arthritis severity in collagen-induced arthritis (CIA) [6]. However, the protective or pro-inflammatory roles of SIRT1 in *in vivo* models of RA have not been analyzed.

Activated myeloid cells, such as monocytes/macrophages, neutrophils, myeloid dendritic cells, and osteoclasts, are known to be prominent participants in inflammatory arthritis such as RA [7]. The activation of nuclear factor-κB (NF-κB) in macrophages results in the transactivation of many pro-inflammatory cytokines and chemokines and leads to RA-FLS activation and the infiltration of large numbers of immune cells into the synovium [8]. Thus, the essential role of NF-κB has been well recognized in the pathogenesis of RA [8], [9]. In a recent report, using a myeloid cell-specific SIRT1 knockout (mSIRT1 KO) mouse model, the deletion of SIRT1 in macrophages led to hyperactive NF-κB signaling, which resulted in enhanced inflammatory signaling in response to environmental stress [10]. However, to our knowledge, no report has assessed the role of myeloid cell-derived SIRT1 in animal models of RA. Thus, to investigate the *in vivo* function of SIRT1 in RA, we generated mSIRT1 KO mice and explored the specific contribution of myeloid cell-derived SIRT1 using the K/BxN serum transfer arthritis model.

## Materials and Methods

### Generation of mSIRT1 KO mice

SIRT1^loxP/loxP^ mice (B6.129-Sirt1^tm1Ygu^/J) and LysM-Cre mice (B6.129P2-lyz2^tm1(cre)Ifo^/J) were purchased from Jackson Laboratory (Bar Harbor, ME, USA). SirT1^loxP/loxP^ and LysM-Cre mice were crossed to obtain mSIRT1 KO mice. To avoid potential variations that could be contributed by gender and genetic background, male mice from the F2 generation, SIRT1^loxP/loxP^LysM-Cre^+/+^ (mSIRT1 KO) and SIRT1^loxP/loxP^LysM-Cre^−/−^ (WT), were used for the studies. All of the experimental animals used in this study were maintained under the protocol approved by the Institutional Animal Care and Use Committee of the Gyeongsang National University (GLA-100917-M0092).

### K/BxN serum transfer arthritis and clinical evaluation

KRN TCR-transgenic mice were a gift from D. Mathis and C. Benoist (Harvard Medical School, Boston, Massachusetts) and from the Institut de Genetique et de Biologie Moleculaire et Cellulaire and were maintained on a B6 background (K/B). To induce K/BxN serum transfer arthritis [11], serum samples were pooled from arthritic adult K/BxN mice. mSIRT1 KO and WT mice were injected with K/BxN serum (50 µL) intraperitoneally on days 0 and 2, and the severity of arthritis was defined as previously described [12]. Clinical arthritis scores were evaluated using a scale of 0–4 for each paw for a total score of 16. Ankle thickness was measured with a caliper placed across the ankle joint at the widest point.

### Histopathological and biochemical analysis

Decalcified and paraffin-embedded joint sections were stained with hematoxylin and eosin for morphological analysis or with a tartrate-resistant acid phosphatase (TRAP) staining kit (Sigma-Aldrich, Hempstead, NY USA) for osteoclast analysis. The joint sections were scored using a semi-quantitative scoring system [12], according to synovial inflammation and bone erosion. The number of TRAP-positive multi-nucleated cells that contained 3 or more nuclei were counted in 10 areas of each ankle (200×, n = 10 mice for each group). The F4/80^+^ cells were immunohistochemically detected with a rat monoclonal antibody directed against F4/80 (Santa Cruz Biotechnology Inc., Santa Cruz, CA, USA). The number of F4/80^+^ cells was counted in 8 randomly selected high-power fields (400×) for each ankle joint (n = 10 mice for each group). The joint tissue concentrations of IL-1β, TNF-α, and IL-6 were measured by ELISA (R&D Systems, Minneapolis, MN, USA).

### Bone marrow-derived macrophage (BMM) culture and differentiation

For the BMM cultures, bone marrow was isolated from the femurs and tibias of WT and mSIRT1 KO mice and cultured in minimum essential medium alpha (α-MEM) supplemented with 10% fetal bovine serum (FBS). The cells were plated and cultured overnight in the presence of macrophage colony-stimulating factor (M-CSF, 10 ng/ml). The non-adherent cells were collected and cultured for 3 days in the presence of M-CSF (10 ng/ml). The floating cells were removed, and the adherent cells were used as BMMs. To assess inflammatory cytokine production, BMMs were pretreated with TNF-α (10 ng/ml) for 24 h. The culture supernatants were collected, and the secreted IL-1β level was quantified by ELISA (R&D Systems). For M1 and M2 differentiation, BMMs were treated with IFN-γ (50 U/ml, Invitrogen) and IL-4 (10 ng/ml; Invitrogen, Carlsbad, CA), respectively.

### Assessment of osteoclast formation

BMMs (5×10^5^) were seeded into 24-well culture plates and maintained in α-MEM-containing recombinant mouse M-CSF (10 ng/ml) and RANKL (10 or 30 ng/ml). After 7 days in culture, the cells were cytochemically stained for TRAP using a commercially available kit (Sigma-Aldrich, Hempstead, NY, USA). The BMMs were rinsed promptly in PBS buffer, fixed with formalin (10% in PBS) for 10 min, and rinsed in distilled water. TRAP was histochemically revealed by a simultaneous coupling reaction using Naphthol AS-BI-phosphate as the substrate and Fast violet B as the diazonium salt. The cells were then incubated for 90 min at 37°C in the dark and rinsed 3 times in distilled water. The residual activity was inhibited by incubation with 4% NaF for 30 min. TRAP-positive cells demonstrating more than 3 nuclei were considered osteoclasts. The number of newly generated osteoclasts was assessed by light microscopic examination.

### BMM migration assay

Murine BMMs (5×10^5^) were plated onto 8-µm pore chemotaxis membranes (Corning, Acton, MA, USA) within Boyden chamber inserts in FBS-free α-MEM. After attachment, MCP-1 (R&D Systems, Minneapolis, MN, USA) was added to the lower chamber, and the cells were incubated at 37°C in 5% CO_2_ for 2 h. The cells that remained on the upper section of the filters were removed mechanically. The cells that migrated to the lower section of the filters were fixed with 4% paraformaldehyde in PBS, stained with DAPI (Invitrogen-Molecular Probes, Eugene, OR, USA), and counted in 10 random fields/filter using an optical microscope (Nikon Co., Tokyo, Japan).

### Western blot analysis

Cells were lysed in RIPA buffer (50 mM Tris, pH 7.2, 150 mM NaCl, 1% NP40, 0.1% SDS) supplemented with a protease inhibitor cocktail (Calbiochem, Cambridge, MA, USA). The cells were then sonicated and centrifuged at 12,000 g for 10 min at 4°C to remove insoluble debris. The protein concentration was determined using the BCA assay ( Thermo Scientific, Waltham, MA, USA). Total proteins (30 µg) were resolved on a SDS-polyacrylamide gel, and the gels were then electroblotted onto a nitrocellulose membrane. After blocking with a 5% skim milk solution, the membranes were incubated with specific antibodies against acetylated p65 (1∶1,000; Cell Signaling Technology, Beverly, MA, USA) or β-actin (1∶5,000; Sigma-Aldrich, Hempstead, NY, USA), and the proteins were identified with the ECL detection system.

### Immunofluorescence assay

BMMs were plated onto a glass coverslip in RPMI 1640 supplemented with 10% FBS and incubated overnight at 37°C. The following day, the cells were treated with TNF-α (10 ng/ml) for 1 h. At the end of the treatments, the cells were washed with PBS, fixed in PBS containing 4% paraformaldehyde, and permeabilized with 0.1% Triton X-100 in PBS for 10 min at room temperature. Then, the cells were blocked with 2% bovine serum albumin (BSA) in PBS for 1 h and incubated with primary monoclonal antibodies against acetylated p65 overnight at 4°C. After washing in PBS, the cells were further incubated with TRITC-conjugated secondary anti-mouse IgG for 1 h. The nuclei were stained with DAPI (0.5 µg/mL; Invitrogen-Molecular Probes). The cover slips were mounted and stored in the dark at 4°C. Fluorescent images were obtained from analyses using the FV-1000 confocal microscope system (Olympus, Tokyo, Japan).

### Electrophoretic mobility shift assay (EMSA)

Nuclear extract preparation and EMSAs were performed as described previously [9]. Nuclear extracts prepared from WT and mSIRT1 KO primary macrophage cells were incubated with a proteinase inhibitor cocktail (Calbiochem, Cambridge, MA, USA) to inhibit endogenous protease activity. An oligonucleotide containing the κ-chain binding site (κB, 5′-CCGGTTAACAGAGGGGGCTTTCCGAG-3′) was synthesized and used as a probe for the gel retardation assay. The 2 complementary strands were then annealed and labeled with [α-^32^P]dCTP. Next, labeled oligonucleotides (10,000 cpm), 10 µg of nuclear extracts, and binding buffer (10 mM Tris-HCl, pH 7.6, 500 mM KCl, 10 mM EDTA, 50% glycerol, 100 ng poly(dI•dC), 1 mM dithiothreitol) were then incubated for 30 min at room temperature in a final volume of 20 µl. The reaction mixtures were analyzed by electrophoresis on 4% polyacrylamide gels in a 0.5× Tris-borate buffer, and the gels were dried and examined by autoradiography. Specificity of the DNA-protein interactions for NF-κB was demonstrated by competition assays using a 50-fold excess of unlabeled oligonucleotide.

### Statistical analysis

Data are expressed as the mean ± SE. Statistical comparisons were performed using 2-tailed Student's t-tests. *P* values less than 0.05 were considered statistically significant.

## Results

### Myeloid SIRT1 deletion leads to the exacerbation of K/BxN serum transfer arthritis

**Figure 1:**
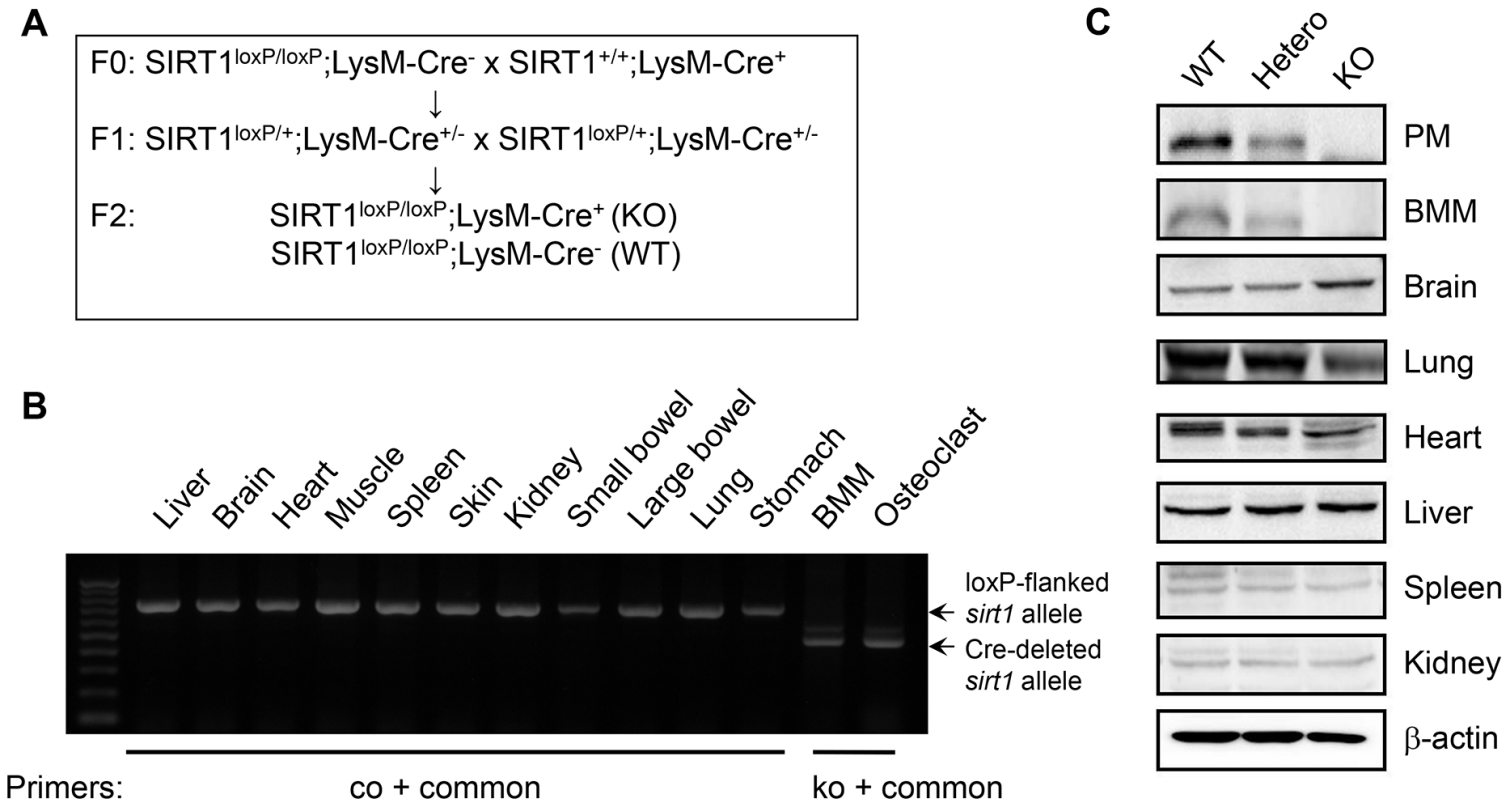
Myeloid cell-specific deletion of SIRT1. (A) Breeding strategy. (B) PCR genotyping. PCR analysis of several tissues from a SIRT1^loxP/loxP^;LysM-Cre^+/+^ mouse was performed using 3 primers designed against sequences upstream and downstream of the loxP-flanked *sirt1* exon 4: 5′ KO primer, 5′-GGTTGACTTAGGTCTTGTCTG; 5′ KO primer, 5′-AGGCGGATTTCTGAGTTCGA; and 3′ common primer, 5′-CGTCCCTTGTAATGTTTCCC. The use of these primers enabled discrimination between the loxP-flanked (upper band) and the Cre-deleted (lower band) *Sirt1* alleles. (C) Western blot analysis for SIRT1 in peritoneal macrophages (PM), BMMs, osteoclasts, and various tissues from SIRT1^loxP/loxP^; LysM-Cre^+/+^ mice.

**Figure 2:**
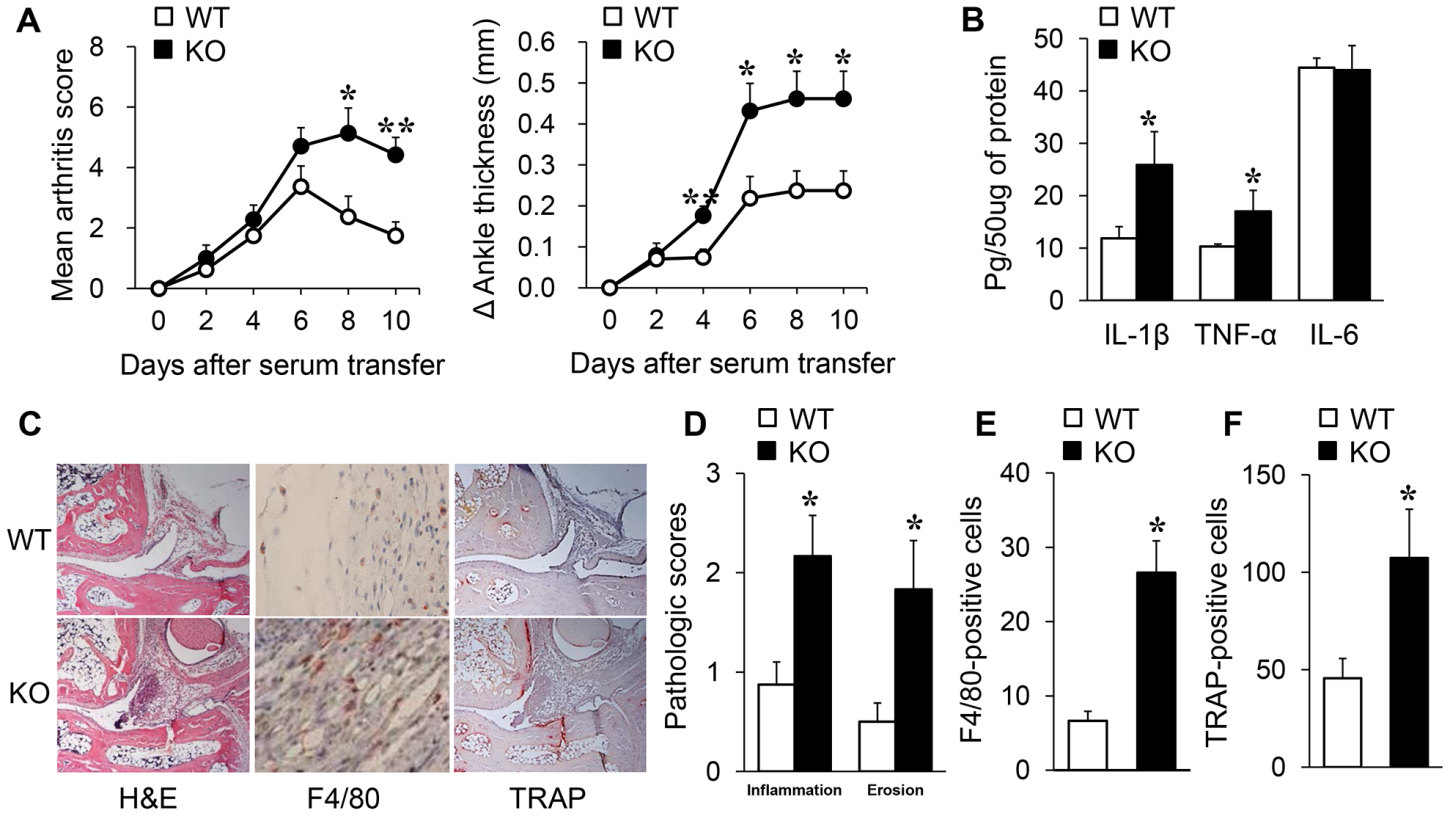
Myeloid cell-specific SIRT1 deficiency aggravates synovial inflammation and bone destruction in K/BxN serum transfer arthritis. Male (8- to 10-week-old) mSIRT1 KO and WT mice were injected with K/BxN serum (50 µL) intraperitoneally on days 0 and 2. (A) The severity of arthritis was defined according to semi-quantitative clinical scoring and the change in ankle thickness. (B) ELISA results for inflammatory cytokines levels in tissue extracts from mouse ankles. (C) Representative sections of the ankle joints stained with H&E, TRAP, and F4/80. The original magnifications are ×100, ×400, and ×100, respectively. (D) Mean pathologic scores for synovial inflammation and bone erosion. (E) F4/80^+^ macrophages in ankle joints and (F) the numbers of TRAP-positive cells. The results of two independent experiments with 10 mice per group are expressed as mean ± SEM, *p<0.05, **p<0.01 vs. WT.

To ascertain the role of the myeloid cell-specific SIRT1 deletion in inflammatory arthritis, we generated mSIRT1 KO mice (Fig. 1) and induced passive K/BxN arthritis in mSIRT1 KO mice and matched littermate control (WT) mice. In the K/BxN serum transfer model, the arthritis severity and change in ankle thickness were significantly increased in mSIRT1 KO mice as compared to WT mice (Fig. 2A). There was no difference in arthritis severity between SIRT1^+/+^LysM-Cre^+/+^ and SIRT1^+/+^LysM-Cre^−/−^ mice (data not shown). It is well known that pro-inflammatory cytokines, such as IL-1β and TNF-α, play key roles in the pathogenesis of RA. Therefore, we measured cytokine levels in the synovial tissues on day 10 using ELISA. The levels of TNF-α and IL-1β were higher in the ankle lysates of mSIRT1 KO mice than WT mice, although the IL-6 levels were not increased (Fig. 2B). Histopathological analysis further revealed that mSIRT1 KO mice demonstrated more severe synovial inflammation and bone erosions than WT mice (Fig. 2C left panel, 2D). Because macrophages are important mediators of innate immunity and are critical in the pathogenesis of inflammatory arthritis, we next examined macrophage infiltration into the synovial tissue. mSIRT1 mice showed increased F4/80^+^ macrophage infiltration in the synovial tissues when compared to WT mice (Fig. 2C middle panel, 2E). We next counted TRAT-positive cells to confirm the relationship between bone erosion and the number of osteoclasts, and TRAP-positive osteoclasts were markedly increased in the joints of mSIRT1 KO mice (Fig. 2C right panel, 2F).

### Myeloid SIRT1 deletion increases the activation of macrophages and osteoclastogenesis

**Figure 3:**
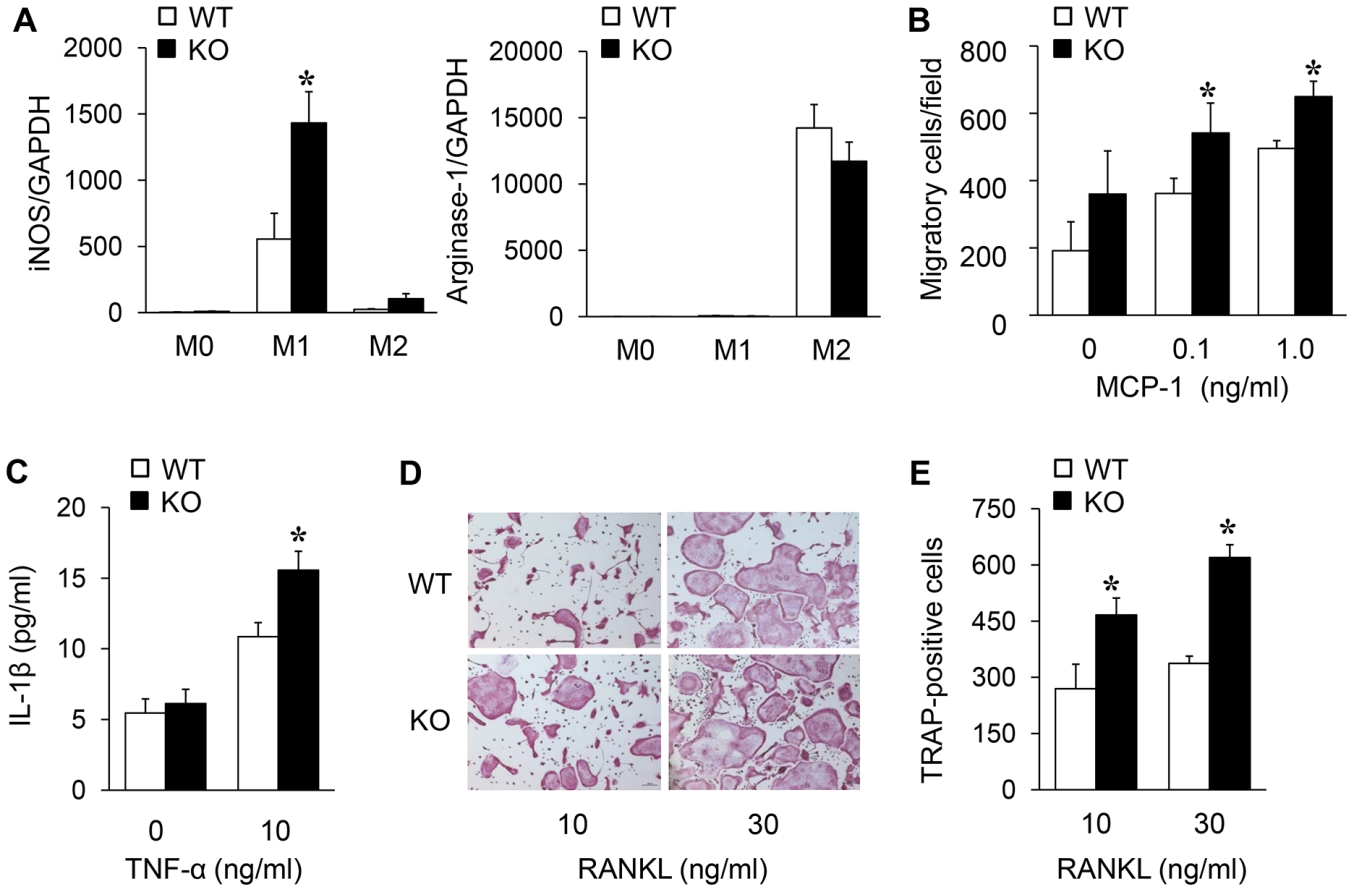
SIRT1 deficiency increases the migration, cytokine release, and osteoclastogenesis of BMMs. (A) Regulation of macrophage polarization by SIRT1. SIRT1-deficient or WT BMMs were treated with either 50 U/ml of IFN-γ (for M1 polarization) or 10 ng/ml of IL-4 (for M2 polarization) for 48 h. (B) Effect of recombinant MCP-1 on the migration of BMMs. (C) Increased release of IL-1β from SIRT1-deficient BMMs following 12-h TNF-α treatment. (D) Microscopic view of TRAP-positive cells from BMM precursors cultured with M-CSF and RANKL. Original magnification, ×100 (E) The TRAP-positive multinucleated osteoclasts in each well were counted. Values  =  mean ± SE, n = 4–5 mice for each group, *p<0.05 and ^**^ *p*<0.01 vs. WT.

In RA, activated macrophages play a central role, and the imbalance of pro-inflammatory and anti-inflammatory macrophages is often observed. The macrophage polarization state can be defined by specific markers, such as iNOS and arginase-1 for cells of the M1 and M2 phenotypes, respectively. Thus, to examine the effect of SIRT1 on macrophage polarization, BMMs from mSIRT1 KO and WT mice were treated with IFN-γ for M1 polarization or IL-4 for M2 polarization. In the M1 polarization condition, iNOS expression was significantly increased in BMMs from mSIRT1 KO mice, whereas the levels of arginase-1 were unaltered in the M2 polarization condition (Fig. 3A). These findings suggest that SIRT1-deleted macrophages display increased M1 polarization. Next, we examined the effect of SIRT1 deletion on macrophage migration. Consistent with the increased number of macrophages in the synovium of arthritic mice, the loss of SIRT1 significantly enhanced MCP-1-induced macrophage migration in a dose-dependent manner (Fig. 3B). We also investigated whether SIRT1 deletion affects cytokine production by macrophages by measuring the IL-1β levels in culture media from TNF-α-stimulated mSIRT1 KO and WT macrophages. The results indicated that SIRT1 deletion increased IL-1β secretion 1.5-fold over control macrophages (Fig. 3C). Furthermore, we assessed osteoclastic differentiation from BMMs in medium supplemented with M-CSF and RANKL to investigate the process leading to increased bone resorption in mSIRT1 KO mice. The absence of SIRT1 resulted in the formation of greater numbers of multinucleated TRAP-positive cells (Fig. 3D, E).

### Myeloid SIRT1 deletion induces the hyperacetylation of p65 and the hyperactivation of NF-κB

**Figure 4:**
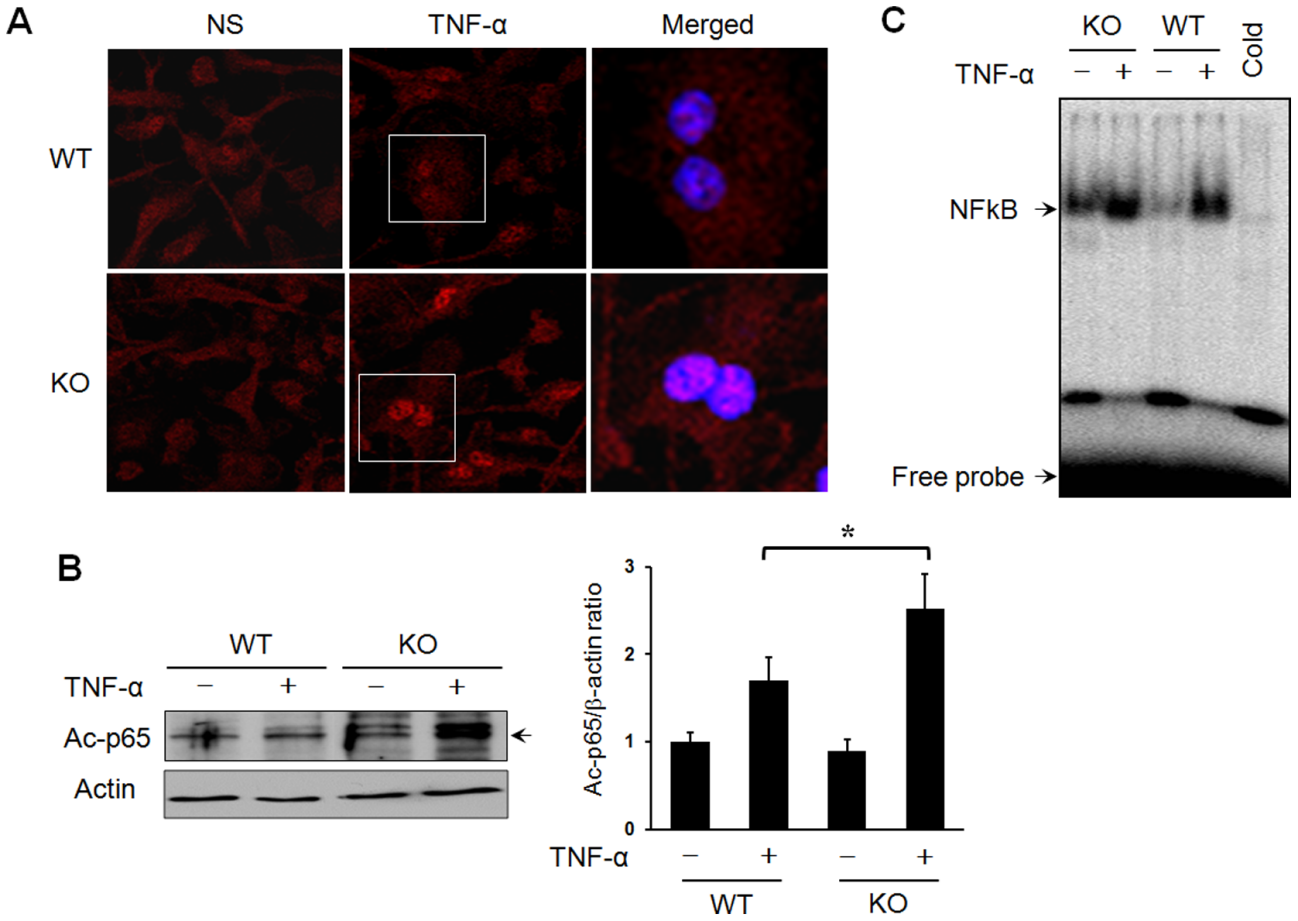
SIRT1 deficiency induces hyperactivation of NF-κB in BMMs. BMMs were cultured from mSIRT1 or WT mice and stimulated with TNF-α (10 ng/ml) for 1 h. (A) Immunostaining for acetylated p65 showed that loss of SIRT1 resulted in higher and sustained levels of acetylated p65 in the nuclei of macrophages following TNF-α treatment. (B) The levels of acetylated p65 were analyzed by Western blotting. Representative blot shown is from one of the three independent experiments with similar results. Values  =  mean ± SE, *p<0.05 vs. WT. (C) The DNA binding activity of NF-κB was analyzed by EMSA.

SIRT1 is known to regulate the transcriptional activity of NF-κB through the direct deacetylation of its p65 subunit at lysine 310 [13]. To determine whether loss of SIRT1 in macrophages alters the acetylation status of p65, BMMs from mSIRT1 KO and WT mice were stimulated with TNF-α and analyzed by immunofluorescent staining and Western blotting using an anti-Ac-K310 antibody. BMMs from both mSIRT1 KO and WT mice displayed low basal levels of Ac-p65, and TNF-α treatment induced the acetylation of p65 in both mSIRT1 KO and WT BMMs. However, mSIRT1 KO BMMs displayed significantly higher levels of Ac-p65 and increased p65 levels in their nuclei (Fig. 4A, B). To test whether this increased acetylation of p65 influenced NF-κB binding activity, mSIRT1 KO and WT BMMs were stimulated with TNF-α, and the nuclear fractions of the cell lysates were analyzed by EMSA. The DNA binding activity to an NF-κB consensus sequence in mSIRT1 KO BMMs was increased before and after TNF-α stimulation as compared to that observed in WT BMMs (Fig. 4C).

## Discussion

In this study, we used mSIRT1 KO mice to investigate the role of SIRT1 in the K/BxN serum transfer model of inflammatory arthritis. Our results provide *in vivo* evidence that myeloid cell-specific deletion of SIRT1 exacerbates inflammation and bone erosion in K/BxN serum transfer arthritis. Furthermore, our *in vitro* results showed that SIRT1-deleted macrophages display increased M1 polarization, migration, pro-inflammatory cytokine production, and osteoclastogenesis through the hyperacetylation and consequent hyperactivation of NF-κB. Together, our findings suggest that SIRT1 plays a protective role against inflammatory arthritis such as RA.

Several animal studies have demonstrated that SIRT1 exhibits pronounced anti-inflammatory properties. Hepatocyte-specific SIRT1-deleted mice challenged with a high-fat diet developed hepatic inflammation and hepatic steatosis [14], and SIRT1-null mice were shown to accumulate deposits of immune complexes in the liver and kidney and develop an autoimmune-like condition [15]. Furthermore, Schug *et al*. showed that ablation of SIRT1 in macrophages not only increased the inflammatory response but also predisposed mice to the development of insulin resistance and metabolic disorders [10], and our data demonstrated that myeloid cell-specific SIRT1 deletion led to enhanced inflammation and bone destruction in K/BxN serum transfer arthritic mice. These findings suggest that myeloid cell-derived SIRT1 may be a negative regulator of the inflammatory response in RA.

The roles of monocytes/macrophages in RA are characterized by their migration, maturation, activation, cytokine production, and interactions with other synovial cells [7]. Here, we demonstrated that macrophages from mSIRT1 KO mice displayed increased migration in both *in vitro* and *in vivo* experiments. It is widely known that TNF-α, IL-l, and IL-6 released by M1 macrophages are abundant in RA, whereas IL-10 activity, which is characteristic of M2 macrophages, is somewhat diminished [7]. Accordingly, we also found that SIRT1-deficient macrophages exhibited increased M1 polarization and increased proinflammatory cytokine production. Zainabadi previously reported that SIRT1-knockout mice displayed significant bone deficiencies associated with increased osteoclastogenesis [16]. Consistent with this finding, we found that the differentiation and infiltration of osteoclasts were increased in SirT1-deficient BMMs *in vitro* as well as K/BxN serum-induced arthritic M-SIRT1 KO mice *in vivo*. Together, these findings indicate that myeloid cell-derived SIRT1 plays a critical role in the control of macrophage migration and activation as well as osteoclastogenesis.

Previous reports have shown that SIRT1 deacetylates p65 and interferes with the NF-κB signaling pathway, thereby acting as an anti-inflammatory factor [10], [17]. Because NF-κB is a molecular target for both the inflammatory response and deacetylation by SIRT1, we investigated changes in the NF-κB signaling pathway in SIRT1-deleted BMMs, and our results showed that the loss of SIRT1 resulted in higher and sustained acetylation of p65 following TNF-α stimulation. We further demonstrated that the DNA binding of NF-κB was increased in M-SIRT1 KO nuclear lysates compared to controls following TNF-α stimulation. Collectively, these data indicate that the absence of SIRT1 leads to hyperacetylation and hyperactivation of NF-κB, which results in the exacerbation of inflammatory arthritis.

In summary, our study provides *in vivo* evidence that myeloid cell-specific deletion of SIRT1 exacerbates inflammatory arthritis. Contrary to previous reports showing a pro-inflammatory role of SIRT1 in synovial cells, our data suggest an anti-inflammatory property of SIRT1 in RA. Recent studies has suggested that, in addition to myeloid cells, suppression of SIRT1 in other immune cells such as in T cells and dendritic cells plays different roles in inflammation [18], [19]. In addition, SIRT1 activators may have therapeutic effects in inflammatory autoimmune disease by blocking macrophage trafficking [20]. Thus, further investigation using chronic CIA model and other cell-specific KO mice will be needed to delineate the protective or harmful effect of SIRT1 on RA pathogenesis as well as potential future therapeutic application in RA.
